# Preparation of Lignocellulose-Based Activated Carbon Paper as a Manganese Dioxide Carrier for Adsorption and *in-situ* Catalytic Degradation of Formaldehyde

**DOI:** 10.3389/fchem.2019.00808

**Published:** 2019-12-09

**Authors:** Xiao Zhang, Chunhui Zhang, Qixuan Lin, Banggui Cheng, Xinxin Liu, Feng Peng, Junli Ren

**Affiliations:** ^1^State Key Laboratory of Pulp and Paper Engineering, South China University of Technology, Guangzhou, China; ^2^School of Light Industry and Engineering, South China University of Technology, Guangzhou, China; ^3^College of Materials Science and Technology, Institute of Biomass Chemistry and Technology, Beijing Forestry University, Beijing, China

**Keywords:** lignocellulose biomass, carbon fiber paper, manganese dioxide, catalytic degradation, formaldehyde

## Abstract

Formaldehyde is a colorless, highly toxic, and flammable gas that is harmful to human health. Recently, many efforts have been devoted to the application of activated carbon to absorb formaldehyde. In this work, lignocellulose-based activated carbon fiber paper (LACFP) loaded with manganese dioxide (MnO_2_) was fabricated for the adsorption and *in-situ* catalytic degradation of formaldehyde. LACFP was prepared by two-stage carbonization and activation of sisal hemp pulp-formed paper and was then impregnated with manganese sulfate (MnSO_4_) and potassium permanganate (KMnO_4_) solutions; MnO_2_ then formed by *in situ* growth on the LACFP base by calcination. The catalytic performance of MnO_2_-loaded LACFP for formaldehyde was then investigated. It was found that the suitable carbonization conditions were elevating the temperature first by raising it at 10°C/min from room temperature to 280°C, then at 2°C/min from 280 to 400°C, maintaining the temperature at 400°C for 1 h, and then increasing it quickly from 400 to 700°C at 15°C/min. The conditions used for activation were similar to those for carbonization, with the temperature additionally being held at 700°C for 2 h. The conditions mentioned above were optimized to maintain the fiber structure and shape integrity of the paper, being conducive to loading with catalytically active substances. Regarding the catalytic activity of MnO_2_-loaded LACFP, the concentration of formaldehyde decreased by 59 ± 6 ppm and the concentration of ΔCO_2_ increased by 75 ± 3 ppm when the reaction proceeded at room temperature for 10 h. The results indicated that MnO_2_-loaded LACFP could catalyze formaldehyde into non-toxic substances.

## Introduction

With the increasing development of the world economy, environmental problems are becoming more and more serious all over the world. Among pollution sources, volatile organic compounds (VOCs) are harmful atmospheric pollutants for human and animals (Zhu et al., 2019). Of these VOCs, formaldehyde, due to its chronic toxicity and extensive sources, has become one of the most pervasive air pollutants (Nie et al., 2016). Formaldehyde is also included in a list of carcinogens published by the International Agency for Research on Cancer (Salthammer, 2013). Therefore, the removal of formaldehyde is necessary for human health.

Catalytic oxidation has been proved to be a useful method for degrading formaldehyde. It mainly includes photocatalytic oxidation (Sun et al., 2010; Dou et al., 2019), plasma technology (Saulich and Müller, 2013; Lo et al., 2015), thermal catalytic oxidation (Liu et al., 2019), and room-temperature catalysis (Wang et al., 2015). However, both photocatalytic oxidation and thermal catalytic oxidation use large-scale equipment, such as a heating unit and a light source. Furthermore, plasma technology requires the use of plasma instruments. These facilities occupy a large amount of space and are high-cost. Thus, many researchers have focused on the catalytic oxidation of formaldehyde at room temperature. There are two main catalytic materials for the catalytic oxidation of formaldehyde at ambient temperature, namely noble metals, such as Pt (Cui et al., 2015; Wang L. et al., 2017), Pd (Huang and Leung, 2011; Tan et al., 2015), Au (Hong et al., 2016; Ma et al., 2016), and Ag (Lu et al., 2018), and non-noble metal oxides, such as MnO_2_ (Dai et al., 2016; Rong et al., 2018), CuO (Jin et al., 2013; Pei et al., 2015), and TiO_2_ (Zeng et al., 2014). Among non-noble metal oxides, MnO_2_ is considered to be an excellent catalyst for the catalytic degradation of formaldehyde.

Activated carbon fiber (ACF) is used to adsorb formaldehyde because of its large specific surface area, abundant micropores, and excellent adsorption capacity (Gopinath and Kadirvelu, 2018; Puziy et al., 2018). However, formaldehyde-adsorbed ACF could cause secondary pollution of the environment. Furthermore, as present, the common precursors used in the preparation of ACF are pitch (Dai et al., 2016), polyacrylonitriles (Lee et al., 2013), and phenolic compounds (An et al., 2009), which originate from fossil fuels. In contrast, biomass-based carbon materials have gained widespread attention because of their abundance, renewability, superior recyclability, and environmental properties. Many efforts have also been devoted to determining how to improve the adsorption capacity of biomass-based carbon materials for formaldehyde (Boonamnuayvitaya et al., 2005; Kumagai et al., 2008; Abdul Manap et al., 2018). For example, Boonamnuayvitaya et al. (2005) prepared activated carbons from coffee residues using the different activators of zinc chloride, nitrogen, carbon dioxide, and steam, of which preparation with zinc chloride showed the highest capacity for formaldehyde adsorption. Kumagai et al. (2008) prepared carbons and activated carbons to remove formaldehyde by using rice husks and found that the activated carbon demonstrated higher adsorption capacity than the carbon preparation. However, secondary pollution is still a challenge (how to treat the formaldehyde-adsorbed biomass-based carbon materials). Therefore, it is important to develop new technologies for the removal of formaldehyde by adsorption and *in situ* catalysis using biomass-based carbon materials loaded with noble metals or metal oxides, which will be favorable to environmental protection.

Herein, lignocellulose-based activated carbon fiber paper (LACFP) loaded with MnO_2_ was fabricated to adsorb formaldehyde and then used for *in-situ* MnO_2_-loaded catalysis to degrade formaldehyde at room temperature. LACFP was prepared by two-stage carbonization and activation of sisal hemp pulp-formed paper so as to obtain a long fiber structure and maintain the shape integrity of the paper. MnO_2_ was then formed by *in situ* growth in the internal structure of the LACFP and on the surfaces of fibers by the impregnation of LACFP with solutions of manganese sulfate (MnSO_4_) and potassium permanganate (KMnO_4_) in turn. LACFP and MnO_2_-loaded LACFP were characterized by thermogravimetry (TG), Fourier transform infrared spectroscopy (FTIR), scanning electron microscopy (SEM), energy dispersion spectrum (EDS), transmitting electron microscopy (TEM), Brunauer–Emmett–Teller (BET), X-ray photoelectron spectroscopy (XPS), and X-ray diffraction (XRD). In addition, the catalytic performance of MnO_2_-loaded LACFP for the degradation of formaldehyde was investigated.

## Experimental Section

### Materials

Pulp board of unbleached sisal hemp was obtained from Zhuzhou Times New Material Technology Co., Ltd, and the contents of cellulose, hemicellulose, and lignin were measured, which were 64.0, 17.7, and 7.0 wt%, respectively. Potassium hydroxide (KOH, AR) was purchased from Guangzhou Chemical Reagent Factory. Potassium permanganate (KMnO_4_, AR) was purchased from Shantou Guanghua Chemical Factory Co., Ltd. Manganese sulfate (MnSO_4_, AR) was purchased from Shanghai Macklin Biochemical Co., Ltd. Formaldehyde (HCHO, AR) solution containing 37% formaldehyde was bought from Hubei Aosheng New Material Technology Co. Ltd. These chemicals were used as received without further purification.

### Preparation of LACFP

The sisal hemp pulp board (dried pulp: 2.8 g) was split into pieces, impregnated in water for 48 h and then highly dispersed at a stirring speed of 6,000 r/min. Subsequently, the dispersed pulp was used to form the paper sheet (quantitation: 140 g/m^2^) using papermaking equipment. The paper sheet was cut into small rectangular pieces of paper (length 100 mm, width 50 mm). The paper sample was placed in a stainless steel horizontal tubular furnace (length 900 mm, interior diameter 800 mm) and carbonized through a certain heating procedure in a nitrogen atmosphere. Importantly, the carbonation was carried out in several stages according to the TGA result for the sisal hemp pulp-based paper. After the carbonation reaction was over, the sample was impregnated with a given concentration of KOH solution for 12 h. The impregnated sample was then dried at 105°C for 5 h. The activation conditions were the same as with the carbonization mentioned above, but the holding time at the activation temperature was increased to 2 h. LACFP was formed and washed with hot ultrapure water several times, then dried in an oven at 105°C for 5 h.

### Loading of LACFP With MnO_2_

MnO_2_ was formed by *in-situ* growth in LACFP. 151 mg of MnSO_4_ and 105 mg of KMnO_4_ were dissolved in 50 ml of ultrapure water and stirred for 1 h, respectively. LACFP was immersed in the MnSO_4_ solution for 12 h. It was then taken out, and the KMnO_4_ solution was added dropwise into the sample with power ultrasound for 30 min. Subsequently, the KMnO_4_ solution including the LACFP was reacted at 90°C for 5 h. The sample was dried at 105°C for 5 h and was calcined at 280°C for 2 h. Consequently, MnO_2_-loaded LACFP was formed.

### Characterization

TG (TAQ500, America) was used to analyze the thermal behavior of sisal hemp pulp-based paper under a nitrogen atmosphere at a specific flow. The first heating procedure was set from 20 to 800°C at a rate of 10°C/min. The FTIR spectrum was recorded with a VERTEX 70 spectrometer (Bruker, Germany). SEM (ZEISS, Germany), with an EDS detector, was used to examine the morphological structure of the LACFP and MnO_2_-loaded LACFP and to determine the chemical compositions of MnO_2_-loaded LACFP. The nitrogen adsorption–desorption isotherms were obtained with a TriStar II 3flex (Micromeritics Instrument, USA) at 77 K. The XRD (Bruker, Germany) patterns of the LACFP and MnO_2_-loaded LACFP were obtained on a Bruker diffractometer with Cu Kα radiation. XPS (Kratos Axis Ulra DLD) was used to analyze the chemical state of Mn in the sample.

### Catalytic Performance of MnO_2_-Loaded LACFP

**Figure 1:**
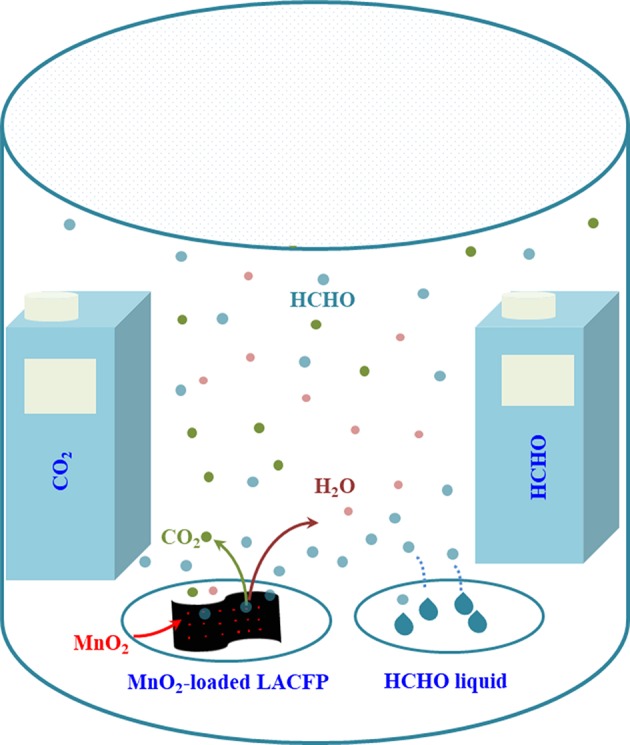
Schematic diagram of the device for catalytic degradation reaction of formaldehyde using MnO_2_-loaded LACFP.

Catalytic degradation of formaldehyde using MnO_2_-loaded LACFP was performed in a sealed glass reactor. MnO_2_-loaded LACFP (100 mg) was placed in a petri dish and settled in the reactor. The formaldehyde solution (10 μL) was injected into another petri dish, which was also placed in the reactor. The detectors for CO_2_ and formaldehyde were placed in this sealed glass bottle, respectively. After the sample, formaldehyde solution, and detectors had been placed in the glass bottle, the adsorption and degradation reaction of formaldehyde started, and the detectors began to detect the concentrations of formaldehyde and CO_2_. The time of every experiment was controlled to 10 h. Meanwhile, a comparative study was carried out using no catalyst and LACFP (100 mg). A portable formaldehyde alarm detector (PGD3-C-CH_2_O) and portable CO_2_ alarm detector (PGD3-C-CO_2_), which were purchased from Shenzhen Xinss Technology Development Co., Ltd, were used to test the concentrations of gaseous HCHO and CO_2_, respectively. The catalytic performance of the sample was evaluated by analyzing the increase in the ΔCO_2_ concentration and the decrease in the formaldehyde concentration. Figure 1 depicts a schematic diagram of the device for catalytic degradation reaction of formaldehyde using MnO_2_-loaded LACFP.

## Results and Discussion

### Analysis of Carbonization Conditions

**Figure 2:**
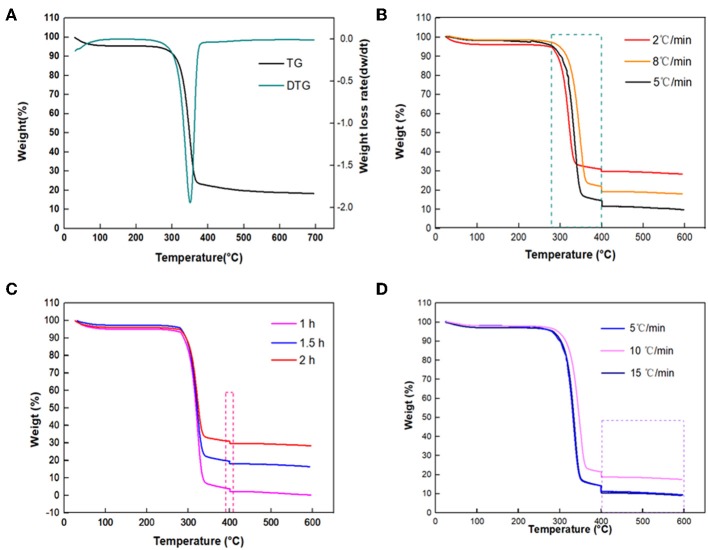
TG and DTG curves of sisal hemp pulp-based paper **(A)**; TG curves at 280–400°C at different heating rates (8, 5, and 2°C/min) **(B)**; at 400°C with different holding times (1, 1.5, and 2 h) **(C)**; and at 400–600°C at different heating rates (5, 10, and 15°C/min) **(D)**.

**Figure 3:**
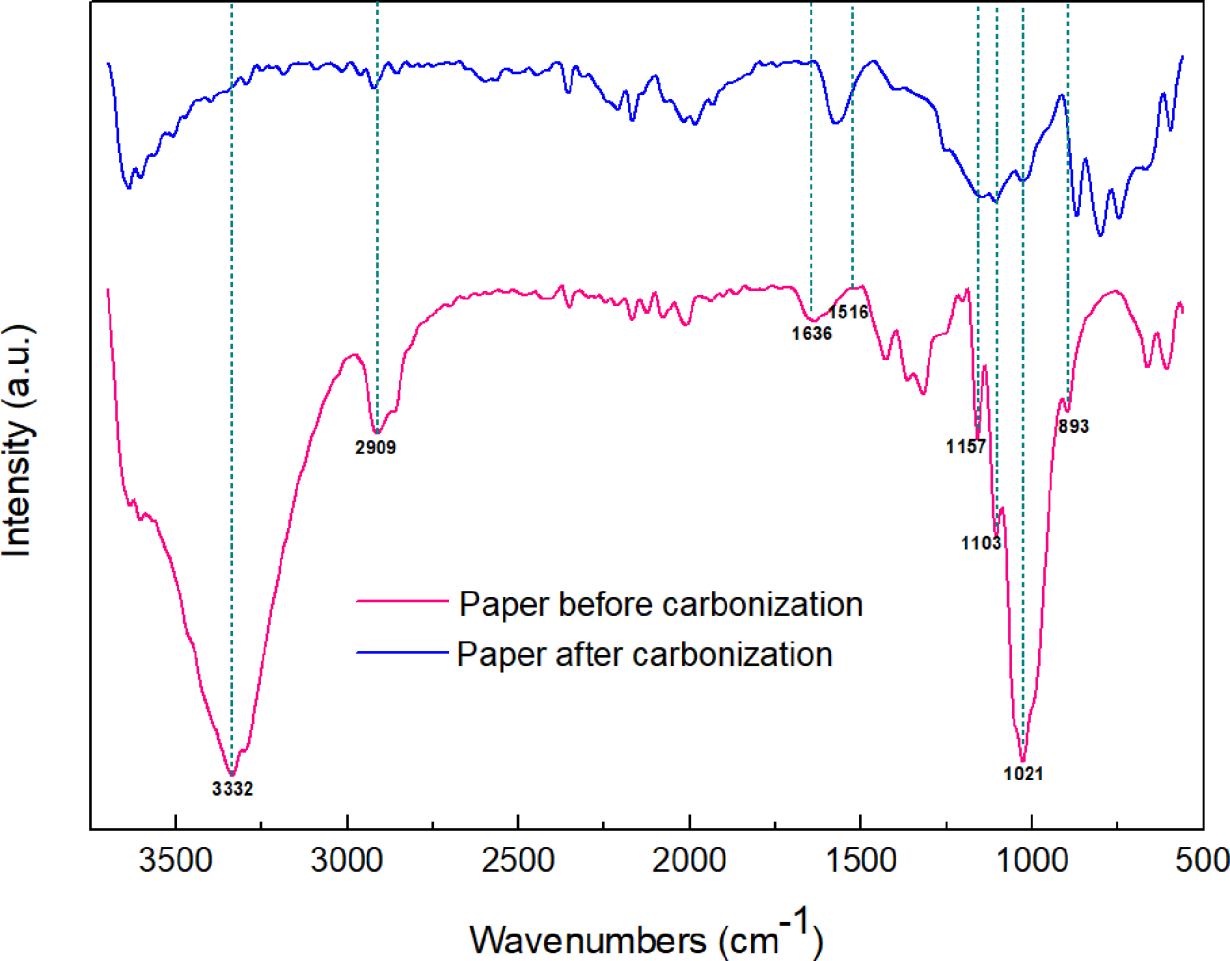
FTIR spectra of sisal hemp pulp-based papers before and after carbonization.

TG and differential thermogravimetry (DTG) curves of sisal hemp pulp-based paper are shown in Figure 2A. The heating procedure was set as going from 20 to 700°C at a heating rate of 10°C/min. Three main weight loss stages could be observed, which occurred in the temperature ranges of room temperature-280°C, 280–400°C, and 400–700°C, respectively. In this study, sisal hemp pulp was applied as the raw material, in which most lignin and pectin were removed by cooking technology, and there was 64.0 wt% cellulose, 17.7 wt% hemicellulose, and 7.0 wt% lignin. Compared to cellulose and lignin, hemicellulose had relatively lower thermal stability. In the TGA of hemicellulose, cellulose, and lignin, the main weight loss of hemicellulose mainly occurred at 220–315°C (Yang et al., 2007), and the main pyrolysis temperature of cellulose was in the range from 315 to 400°C (Yan Y. et al., 2018), whereas, compared with hemicellulose and cellulose, lignin had high thermal stability and could be decomposed from 400 to 500°C (Arias et al., 2006; Ishak et al., 2011; Chen et al., 2019). For Figure 2A, considering the content of the components of sisal hemp pulp and thermal stability of the three main components, the content of hemicellulose in the sisal hemp pulp-based paper was only 17.6%, so analysis of the DTG shows no large mass loss from room temperature to 280°C. The pyrolysis occurring from room temperature to 280°C mainly included decomposition of hemicellulose and removal of physically adsorbed water. A sharp mass loss step occurred beginning at 280°C and ending at 400°C, with a maximum weight loss rate at 350°C, caused by the decomposition of cellulose in the high-cellulose-content sisal hemp pulp-based paper. After 400°C, the curves of TG and DTG both reached a balanced state, and there was no obvious mass loss. Furthermore, the band at 3,332 cm^−1^ in the FTIR spectra in Figure 3 was attributed to O–H stretch and that at 2,909 cm^−1^ to the stretch of –CH_3_ (Deng et al., 2016). The absorbance at 1,636 cm^−1^ characterizes the absorbed water (Peng and Wu, 2010). The band at 1,516 cm^−1^ was attributed to skeletal vibrations of lignin (Cheng et al., 2018). The characteristic absorption peaks of cellulose and hemicellulose were at 1,157, 1,103, 1,024, and 893 cm^−1^ (Peng and Wu, 2010; Liang et al., 2018). However, it was observed that the characteristic absorption peaks of cellulose and hemicellulose declined and the peaks of lignin basically disappeared after carbonization. According to the results mentioned above, attention should be paid to the heating rate between 280 and 400°C (corresponding to a large mass loss) to optimize the carbonization conditions for obtaining high yields of carbon fibers. Some of the literature has also indicated that the heating rate and holding time both influenced the morphology and structure of the sample (Sangmanee and Maensiri, 2009; Pan et al., 2011). In this work, different heating rates of 8, 5, and 2°C /min for carbonization temperatures from 280 to 400°C were comparatively investigated to minimize the mass loss of sisal hemp pulp-based paper as much as possible and maintain fiber length and pore structure.

Figure 2B and Table 1 show that when the heating rates were 8, 5, and 2°C/min, the corresponding mass losses at 280–400°C were 75.33, 81.15, and 63.50%, respectively, which indicated that the mass loss between 280 and 400°C was mainly through the decomposition of most of the cellulose. A heating rate of 2°C/min resulted in the lowest mass loss of the different heating rates. In general, a high heating rate caused larger mass loss and more severe damage to the fiber. Accordingly, 2°C/min is the most desirable heating rate to obtain a high yield of carbon fibers.

**Table 1:** Mass losses of sisal hemp pulp-based paper at 280–400°C at different heating rates (8, 5, and 2°C/min).

**Heating rate (**°**C/min)**	**Mass loss (%)**
8	75.33
5	81.15
2	63.50

Herein, the influence of the holding time on the mass loss was also studied; the results are shown in Figure 2C and Table 2. When the holding times were 1.0, 1.5, and 2.0 h, the mass losses at 400°C were 1.475, 1.490, and 1.250%, respectively, implying a slight difference. To reduce cost and energy consumption for the preparation of carbon fiber, the suitable condition was deemed to be holding at 400°C for 1.0 h.

**Table 2:** Mass losses of sisal hemp pulp-based paper at 400°C with different holding times (1.0, 1.5, and 2.0 h).

**Holding time (h)**	**Mass loss (%)**
1.0	1.475
1.5	1.490
2.0	1.250

In addition, the effects of heating rates on the mass loss of sisal hemp pulp-based paper between 400 and 600°C at different heating rates were also investigated, as shown in Figure 2D and Table 3. When the heating rates were 5, 10, and 15°C/min, the mass losses in the temperature range of 400–600°C were 1.77, 1.24, and 1.20%, respectively, indicative of a slight difference. This result was consistent with the above TGA in Figure 2A, indicating that the sisal hemp pulp-based paper contained less lignin. Therefore, 15°C/min was used as the heating rate from 400 to 600°C for rapid heating.

**Table 3:** Mass losses of sisal hemp pulp-based paper at 400–600°C at different heating rates (5, 10, and 15°C/min).

**Heating rate (**°**C/min)**	**Mass loss (%)**
5	1.77
10	1.24
15	1.20

**Figure 4:**
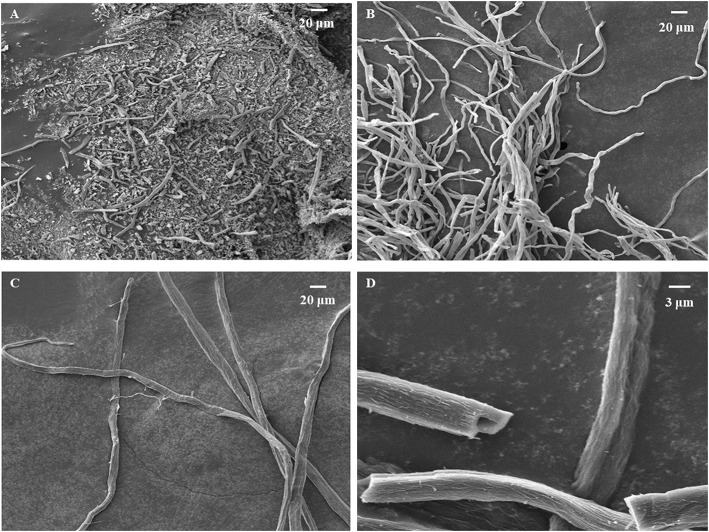
SEM images of carbonized sisal hemp pulp-based paper at 900°C **(A)**, 800°C **(B)**, and 700°C **(C)** and a magnified image of 700°C **(D)**.

**Figure 5:**
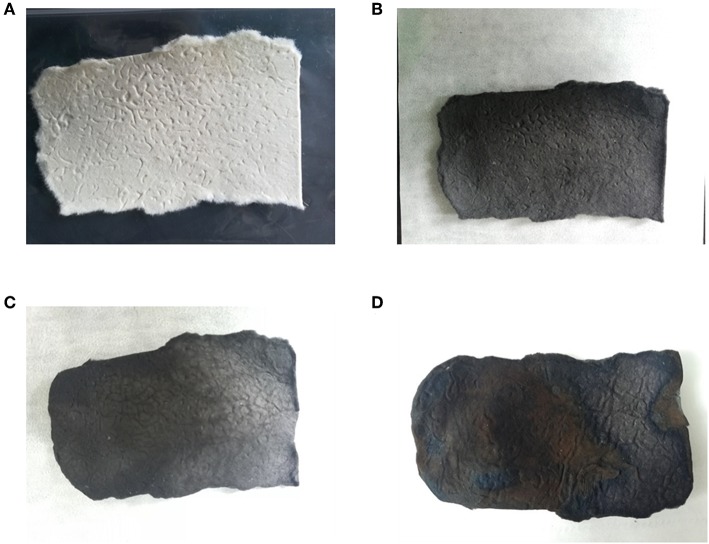
Photographs showing the sisal hemp pulp-based paper before carbonization **(A)**, after carbonization **(B)**, after activation **(C)**, and after MnO_2_ loading **(D)**.

The surface morphology of carbonized sisal hemp pulp-based paper is shown in Figure 4. When the carbonization temperature rose to 800 and 900°C, the structure of the carbon fiber was severely damaged, especially at 900°C (Figures 4A,B). When the carbonization temperature was 700°C, fibers retained their complete structure (Figure 4C), and the cell lumen of the fibers was retained (Figure 4D), which were conducive to preparing adsorbent materials and use as catalyst material carriers. Consequently, the final carbonization temperature used was 700°C. According to the results of TGA, FTIR, and SEM above, the optimal carbonization was conducted as follows: a heating rate of 10°C/min from ambient temperature to 280°C, then at 2°C/min between 280 and 400°C, a holding time of 1 h at 400°C, and then heating at 15°C/min from 400 to 700°C. Photographs of the sisal hemp pulp-based paper before and after carbonization are shown in Figure 5. After carbonization, the area of the paper shank to 52.1% of the original, and the color became black (Figures 5A,B).

### Analysis of Activation Conditions

**Figure 6:**
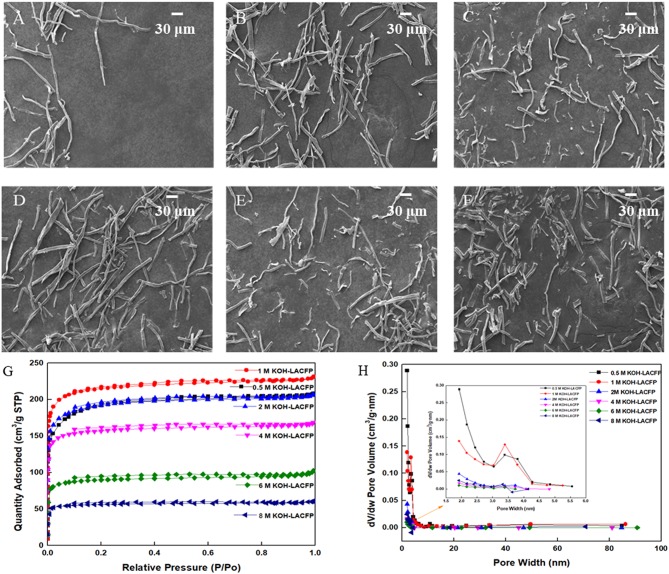
SEM images of LACFP of KOH concentrations at **(A)** 0.5, **(B)** 1, **(C)** 2, **(D)** 4, **(E)** 6, and **(F)** 8 mol/L and their N_2_ adsorption-desorption isotherms **(G)** and pore size distribution curves **(H)**.

In order to increase the specific surface area and enhance the adsorption capacity, sisal hemp pulp-carbon paper was further activated. For activation, the procedure was the same as for the carbonization. However, when the temperature of activation had risen to 700°C, the holding time was increased to 2 h to improve activation efficiency (Aber et al., 2009). In this part, KOH solution was used as the activator, with various concentrations investigated: 0.5, 1.0, 2.0, 4.0, 6.0, and 8.0 mol/L. In the SEM images in Figures 6A–F, it can be seen that with an increase in the activator concentration, the fiber structure was gradually destroyed, which was not conducive to adsorption performance. Generally speaking, a large specific surface area would be beneficial to the catalytic activity of the sample on account of providing more active sites (Li et al., 2014). In addition, N_2_ adsorption–desorption measurements at 77 K were performed to obtain the specific surface area, pore volume, and pore size distribution, which are shown in Figures 6G,H; the detailed data is displayed in Table 4. As the concentration of the KOH solution increased, the total volume and micropore specific surface areas first increased and then decreased, reaching their maximum values at 1 mol/L. The corresponding micropore area and volume were 842.11 m^2^/g and 0.299 cm^3^/g. Micropores accounted for 91.0% of the specific surface area and 84.2% of the pore volume. This was attributed to the fact that a low-concentration KOH solution could not fully activate the carbon fiber. With increasing KOH concentration, the carbon fiber could attain a higher level of activation. However, an excessive amount of KOH caused serious damage to the fiber structure and the collapse of micropores, leading to a decrease in micropore area and specific surface area (Aber et al., 2009). Therefore, a KOH solution of 1 mol/L was suitable as the activator.

**Table 4:** BET results for LACFP using different KOH concentrations.

**KOH concentration** ** (mol/L)**	**S_**BET**_** ** (m^**2**^/g)**	**Micropore area** ** (m^**2**^/g)**	**Total volume** ** (cm^**3**^/g)**	**Micropore volume** ** (cm^**3**^/g)**	**D_**p**_** ** (nm)**
0.5	721	690	0.321	0.290	2.74
1	842	766	0.355	0.299	3.19
2	735	595	0.319	0.235	2.72
4	625	565	0.259	0.216	3.04
6	350	317	0.156	0.124	4.27
8	225	206	0.093	0.0790	3.43

According to the results for carbonization and activation, the specific carbonization and activation procedures of preparing LACFP were as follows: a heating rate of 10°C/min from ambient temperature to 280°C, then at 2°C/min from 280 to 400°C, a holding time of 1 h at 400°C, and heating at 15°C/min from 400 to 700°C for the carbonization process, and a heating rate of 10°C/min from ambient temperature to 280°C, then 2°C/min from 280 to 400°C, a holding time of 1 h at 400°C, heating at 15°C/min from 400 to 700°C, and a holding time of 2 h at 700°C for the activation process. The concentration of KOH for activation was 1 mol/L. The LACFP was prepared successively according to the above procedure (Figure 5C). The prepared LACFP was then used as the carrier for MnO_2_ loading. Table 5 shows the physical parameters of the sisal hemp paper before and after carbonization and after activation. It was found that sisal hemp pulp-based paper had high values of thickness, density, and softness. After carbonization or activation, the corresponding values were decreased to a great extent, which could be due to the mass loss of cellulose, hemicellulose, and lignin during the carbonization process and the formation of holes during the activation process. It is notable that the smaller the softness value, the softer the sample. Thus, the MnO_2_-loaded LACFP had a certain softness performance and was light, indicative of easy processing. Thus, LACFP could be applied as the filtration material of masks in the future.

**Table 5:** Physical parameters of sisal hemp paper before and after carbonization and after activation and MnO_2_ loading.

**Paper**	**Thickness** ** (μm)**	**Density** ** (g/cm^**−3**^)**	**Softness** ** (mN)**
Before carbonization	640	0.22	2,457
After carbonization	295	0.13	224
After activation	268	0.13	109
After MnO_2_ loading	366	0.19	255

### Characterization Analysis of LACFP and MnO_2_-Loaded LACFP

**Figure 7:**
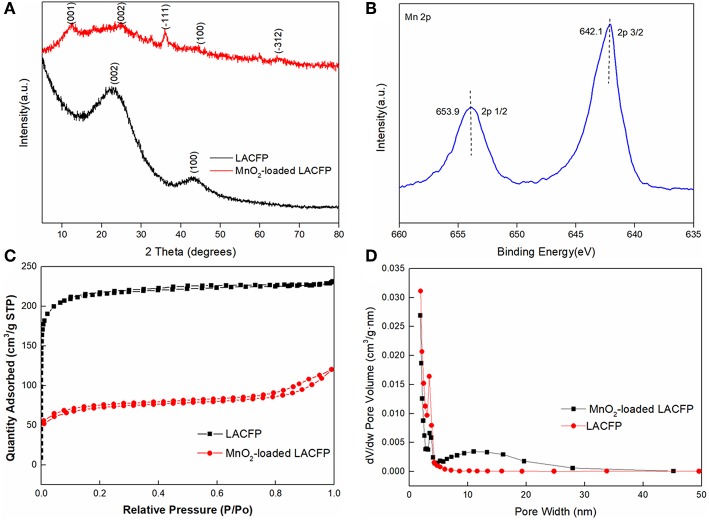
Characterization of LACFP and MnO_2_-loaded LACFP [XRD patterns **(A)**; Mn 2p XPS spectra **(B)**; N_2_ adsorption–desorption isotherms obtained at 77 K **(C)**; and size distribution **(D)**].

The LACFP retained its integrity after loading with MnO_2_, as shown in Figure 5D. Furthermore, its values of thickness, density, and softness increased due to the generation of MnO_2_ (Table 5). However, these values were still much lower than that of sisal hemp paper before carbonization. Figure 7A shows the XRD patterns of LACFP and MnO_2_-loaded LACFP. The two broad peaks at 22.8 and 43, corresponding to the (002) and (100) crystal planes of carbon, are typical diffraction peaks of disordered graphite (Wang et al., 2014; Huang et al., 2015). After LACFP was impregnated with MnSO_4_ and KMnO_4_, respectively, it generated different diffraction peaks at 12.1, 24.5, 36.1, and 64.8, which were ascribed to the (001), (002), (−111), and (020) crystal facets, respectively, and are characteristic peaks of δ-MnO_2_ according to JCPDS 80-1098 (Zhang et al., 2015; Zou et al., 2019). Moreover, all the peaks of δ-MnO_2_ in the composite are in accordance with those of pure MnO_2_. These results implied that MnO_2_ was successfully formed and loaded onto the LACFP.

**Figure 8:**
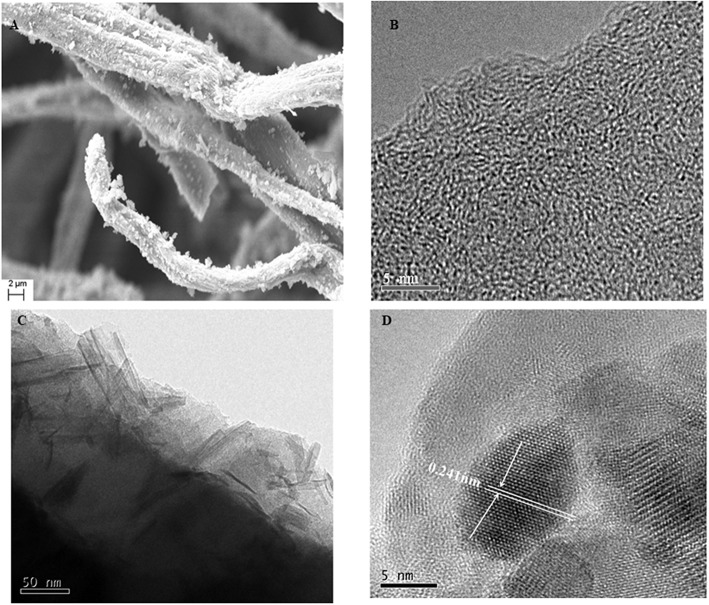
The morphology of LACFP by HRTEM **(B)** and the morphology of MnO_2_-loaded LACFP by SEM **(A)**, TEM **(C)**, and HRTEM **(D)**.

Figure 8 illustrates the morphology of the LACFP and MnO_2_-loaded LACFP as shown by SEM, TEM, and HRTEM. Compared with the image of LACFP (Figure 6B), the surface of LACFP after MnO_2_ loading became rougher, and Figure 8A shows that the MnO_2_ catalyst was loaded on the surface of the LACFP evenly. As we can see from the image in Figure 8B, the lattice fringe in LACFP was unordered. However, after loading with MnO_2_, the TEM and HRTEM images in Figures 8C,D clearly show the existence of the lattice. The dark area may be MnO_2_, because its density is higher than that of carbon, which implies that MnO_2_ was embedded into the LACFP (Dong et al., 2006). The spacing between lattice stripes was 0.241 nm, corresponding to the MnO_2_ (−111) crystal face. This result was consistent with the XRD analysis. The EDS pattern of MnO_2_-loaded LACFP and the contents of C, O, Mg, Si, Cl, K, Ca, and Mn in the sample are displayed in Figure S1. The Mg, Cl, and Ca may come from tap water, which was used to disperse the sisal hemp pulp and form the paper sheet. Si and K may come from ash in the sisal hemp pulp and KMnO_4_, respectively. In general, the EDS spectrum of MnO_2_-loaded LACFP showed that the sample was mainly composed of the elements C, O, and Mn. According to the content of Mn (8.00 ± 0.07%), we calculated the loading amount of MnO_2_ in LACFP as 12.66 ± 0.11%.

XPS analysis was performed to investigate the surface electronic state of the MnO_2_-loaded LACFP. The spectra are showed in Figure 7B and exhibited two main peaks at 642.1 and 653.9 eV, belonging to Mn 2p3/2 and Mn 2p1/2, respectively. Their binding energy difference was 11.8 eV, and the spin-orbit splitting of Mn 2p is 11.7 eV. That indicated that it was close to the MnO_2_ structure (642.2 + 11.7 eV) (Wang J. L. et al., 2017).

The N_2_ adsorption–desorption isotherms operating at 77 K for LACFP and MnO_2_-loaded LACFP are shown in Figures 7C,D. The pore size distribution of LACFP was mainly from 2 to 10 nm, and its peak pore size was 3.39 nm. However, the pore size distribution of MnO_2_-loaded LACFP was wider, from 2 to 45 nm, and its peak pore size was 3.47 nm. Moreover, the adsorption amount of MnO_2_-loaded LACFP considerably declined compared with that of LACFP. Previous studies reported that the pore diameter distribution of MnO_2_ was 11–14 nm (Zhang et al., 2015), whereas the pore diameter distribution of LACFP was 2–10 nm. Therefore, prepared MnO_2_ covered the surface and obstructed and destroyed the pores of LACFP (Wang et al., 2014). The specific surface area and pore volume of MnO_2_-loaded LACFP were 227.13 m^2^/g and 0.165 cm^3^/g. The results indicate that MnO_2_ loading led to an increase in pore size and a decrease in specific surface area compared with LACFP.

### Catalytic Performance

**Figure 9:**
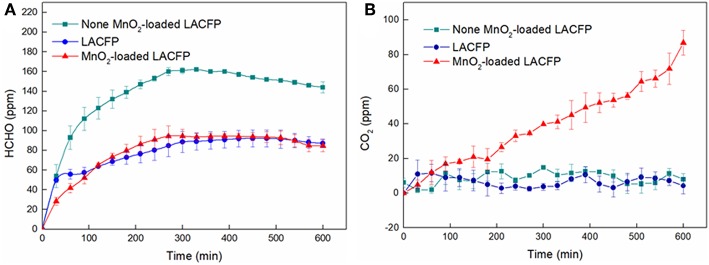
Concentration variation of formaldehyde **(A)** and ΔCO_2_ **(B)** for none^a^, LACFP, and MnO_2_-loaded LACFP (^a^None denotes only formaldehyde solution without other substances).

The catalytic performances of LACFP and the MnO_2_-loaded LACFP are evaluated in Figure 9. The differences in formaldehyde and ΔCO_2_ were used to analyze the catalytic performance of these samples and are given in Table 6. For the control sample, the concentration of formaldehyde increased from 0 to 161 ± 5 ppm in the initial 5 h. However, the concentration of formaldehyde slightly decreased from 161 ± 5 to 144 ± 2 ppm in the second 5 h. The concentration of ΔCO_2_ was in a fluctuating state at 0 due to instrument accuracy effects. The reasons for the decrease in formaldehyde were that the concentration value of formaldehyde was highest after it was fully volatilized, and it took a long time for the formaldehyde to become distributed uniformly. For LACFP, the concentration of formaldehyde increased from 0 to 87 ± 2.5 ppm in the initial 5 h and tended toward stability in the second 5 h, without emitting CO_2_, which implied that formaldehyde was only adsorbed by the LACFP. When the reaction was performed beyond 5 h for MnO_2_-loaded LACFP, the concentration of formaldehyde was nearly unchanged, which suggested that the adsorption for formaldehyde had halted due to saturation in 5 h. In addition, the generation of CO_2_ was about 75 ± 2 ppm for MnO_2_-loaded LACFP, implying that the synergistic effect of LACFP and MnO_2_ led to the adsorption and catalysis of formaldehyde into CO_2_ and H_2_O. Furthermore, the actual value of CO_2_ was a little higher than the theoretical value due to the release of adsorbed formaldehyde in the inner wall and oxidization of formaldehyde into CO_2_, as well as the accuracy of the detector (Yan Z. et al., 2018).

**Table 6:** The values of formaldehyde and ΔCO_2_ over three different samples when the reaction was finished.

**Samples**	**Formaldehyde** ** (ppm)**	**ΔCO_**2**_** ** (ppm)**	**Decreased formaldehyde** ** (ppm)**
None	144 ± 2	0	–
LACFP	87 ± 3	0	56 ± 3
MnO_2_-loaded LACFP	85 ± 5	75 ± 3	59 ± 6

The previous study indicated that the degradation mechanism of formaldehyde can be summarized as an adsorption-degradation-desorption process (Zhou et al., 2011). Is has also been noted that the removal of formaldehyde can include adsorption and oxidation processes (Yan Z. et al., 2018). In the first 3 h of the reaction, adsorption played an important role during the formaldehyde removal process, accompanied by a large amount of formaldehyde adsorption and a small amount of CO_2_ generation. After that, adsorbed formaldehyde quickly began to be oxidized by surface active oxygen into dioctyl maleate (DOM), formate, carbonate, and CO_2_, resulting in a fast decline in the formaldehyde concentration on the sample and large amounts of CO_2_ generation, which indicated that formaldehyde was completely decomposed into CO_2_ (Wang J. L. et al., 2017; Yan Z. et al., 2018; Yan et al., 2019). Besides, the MnO_2_ catalyst can have various crystal structures, namely α, β, γ, λ, and δ structures, which also affect the catalytic activity of MnO_2_ for the oxidation of formaldehyde (Devaraj and Munichandraiah, 2008). Of the α, β, γ, and δ structures, δ-MnO_2_ enhanced the catalytic oxidation of formaldehyde due to its 2D layered structure (Zhang et al., 2015; Wang J. L. et al., 2017). The prepared MnO_2_ on the LACFP was also proved to have the δ structure by XRD analysis. In general, LACFP played the role of adsorbent and carrier, and MnO_2_ provided catalytic sites for catalysis of formaldehyde. MnO_2_-loaded LACFP was thus a kind of portable and catalytic paper that has cost-effective and efficient feasibility for application in the field of the environmental treatment.

## Conclusions

Sisal hemp pulp-based activated carbon fiber paper loaded with MnO_2_ was successfully prepared for the adsorption and *in-situ* catalytic degradation of formaldehyde into CO_2_ and H_2_O. During the carbonization procedure, the heating rate and temperature were important factors controlling the damage level of fibers. In order to maintain the integrity of the fiber and paper shape, the carbonization conditions were optimized. It was found that in the decomposition temperature range corresponding to major mass loss, a low heating rate resulted in a low mass loss of sisal hemp pulp-based paper. The suitable carbonization conditions were heating at 10°C/min from room temperature to 280°C, then at 2°C/min from 280 to 400°C, holding at 400°C for 1 h, and then heating at 15°C/min from 400 to 700°C. The conditions of activation were almost the same as for carbonization, except that there was additional holding at 700°C for 2 h. After loading with MnO_2_ using *in situ* synthesis, the LACFP still retained its shape integrity and some flexibility. MnO_2_-loaded LACFP displayed excellent catalytic performance for formaldehyde. The concentration of formaldehyde decreased by 59 ± 6 ppm and the concentration of ΔCO_2_ increased by 75 ± 3 ppm when the reaction proceeded at room temperature for 10 h. MnO_2_-loaded LACFP has potential for application in toxic gas treatment.

## Data Availability Statement

The raw data supporting the conclusions of this article will be made available by the authors, without undue reservation, to any qualified researcher.

## Author Contributions

XZ and BC designed and carried out the preparation and characterization analysis of LACFP and wrote the research paper. QL and XL carried out the characterization and analysis of catalytic performance of MnO_2_-loaded LACFP and wrote the research paper. JR, CZ, and FP supervised the project, helped design the experiments, evaluated the data, and wrote the manuscript. The results of the manuscript were discussed by all authors.

### Conflict of Interest

The authors declare that the research was conducted in the absence of any commercial or financial relationships that could be construed as a potential conflict of interest.
