# Supplementary material for: Preparation of Lignocellulose-Based Activated Carbon Paper as a Manganese Dioxide Carrier for Adsorption and in-situ Catalytic Degradation of Formaldehyde
Source: Front Chem. 2019 Dec 9;7:808. doi: 10.3389/fchem.2019.00808 (PMC6913189; doi:10.3389/fchem.2019.00808)
Supplement: Supplementary file 1 [file Data_Sheet_1.docx]

Supplementary Material

**Preparation of Lignocellulose-based Activated Carbon Paper as a Manganese Dioxide Carrier for Adsorption and In-situ Catalytic Degradation of Formaldehyde**

**Xiao Zhang^1^, Chunhui Zhang^2,*^, Qixuan Lin^1^, Banggui Cheng^1^, Xinxin Liu^1^, Feng Peng^3^, Junli Ren^1,^***

^1^State Key Laboratory of Pulp and Paper Engineering, South China University of Technology, Guangzhou, 510640, Guangdong, China

^2^School of Light Industry and Engineering, South China University of Technology, Guangzhou, 510640, Guangdong, China

^3^Institute of Biomass Chemistry and Technology, College of Materials Science and Technology, Beijing Forestry University, Beijing, 100083, China

*** Correspondence:**Corresponding Author
E-mail address: chunhui@scut.edu.cn and [renjunli@scut.edu.cn](mailto:renjunli@scut.edu.cn)


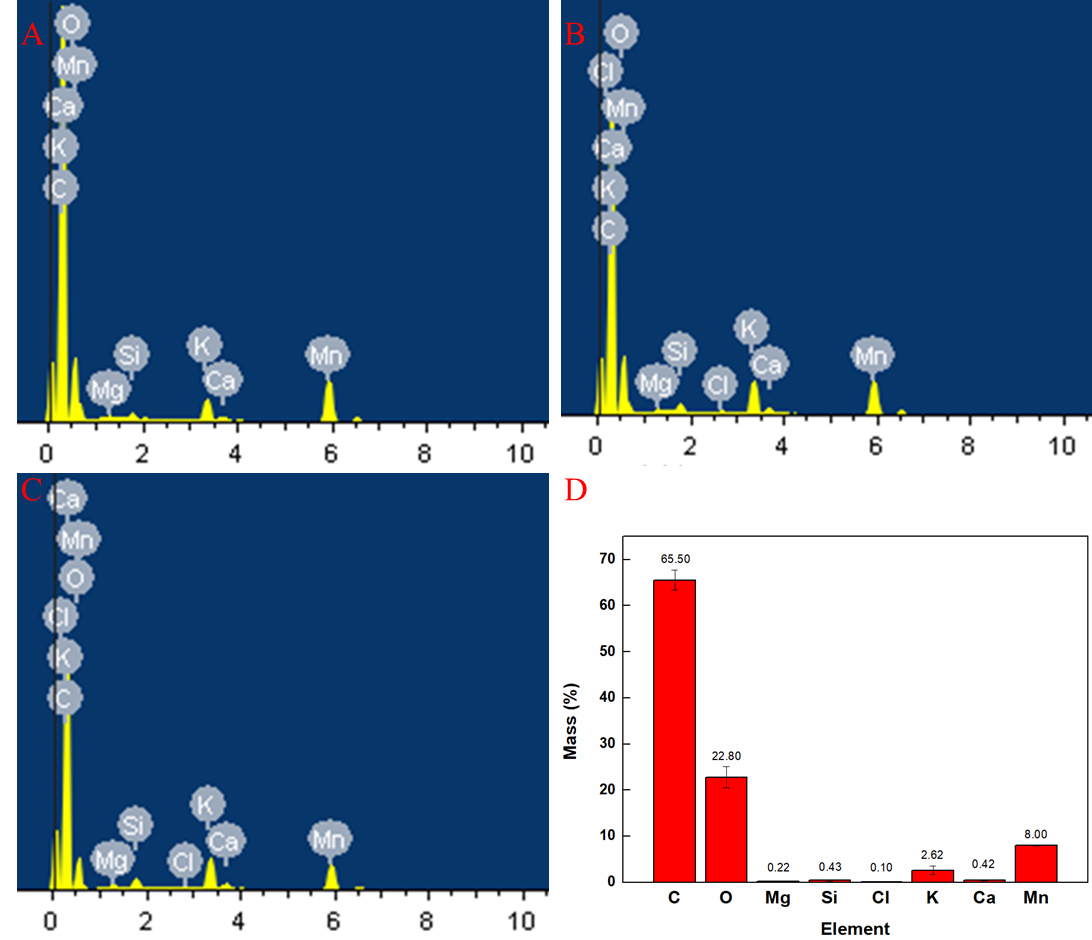


**FIGURE S1** The EDS patterns (A, B and C) of loaded-MnO_2_ LACFP and contents (D) of C, O, Mg, Si, Cl, K, Ca and Mn in the sample.

The MnO_2_ content was calculated according to the following formula:

MnO_2_ wt% = Mn wt% × $\frac{86.94}{54.94}$ （1）

where 86.94 and 54.94 are the molar weights of MnO_2_ and Mn, respectively.
